# Association of Oral health and Genetic Risk with Incidence of Dementia

**DOI:** 10.1093/geroni/igaf122.3885

**Published:** 2025-12-31

**Authors:** Yunrui Liu, Xiang Qi, Ruotong Mona Liu, Sophie Zhijing Xu, Huabin Luo, Bei Wu

**Affiliations:** New York University, New York, New York, United States; New York University, New York, New York, United States; New York University, New York, New York, United States; New York University, New York, New York, United States; East Carolina University, Greenville, North Carolina, United States; NYU Shanghai, Shanghai, China (People’s Republic)

## Abstract

Genetic predisposition and oral health problems both contribute to the risk of dementia, yet it remains unclear whether poor oral health confers similar risk across different levels of genetic susceptibility. We investigated the joint effect of oral health problems (painful gums, bleeding gums, loose teeth, toothache, and dentures) and genetic risk on incident dementia in the UK Biobank cohort. Participants (N = 364,557) free of dementia at baseline (2006–2010) were followed until 2022. Genetic risk was defined by polygenic risk score (PRS; categorized as low, intermediate, and high) and APOE ε4 carrier status. Cox proportional hazards and competing risk regression models were used to estimate associations with all-cause dementia (ACD) and its major subtypes, Alzheimer’s disease (AD) and vascular dementia (VaD), adjusting for demographic, socioeconomic and health-related factors. During follow-up, 1,915 cases of ACD, 3,278 AD, and 1,581 VaD were identified. Oral health problems, particularly painful gums and dentures, were associated with increased risk of ACD, AD, and VaD. Higher genetic risk, assessed by both PRS and APOE ε4, was also strongly associated with incident dementia. Analyses of joint effects indicated that painful gums and dentures demonstrated additive interactions with genetic risk, although no multiplicative interaction was observed. These findings suggest that poor oral health is independently associated with elevated dementia risk and may exacerbate the burden among genetically susceptible individuals. Improving oral health care could represent a modifiable strategy for dementia prevention.

